# Development and evaluation of a real-time RT-PCR and a field-deployable RT-insulated isothermal PCR for the detection of Seneca Valley virus

**DOI:** 10.1186/s12917-019-1927-4

**Published:** 2019-05-24

**Authors:** Jianqiang Zhang, Charles Nfon, Chuan-Fu Tsai, Chien-Hsien Lee, Lindsay Fredericks, Qi Chen, Avanti Sinha, Sarah Bade, Karen Harmon, Pablo Piñeyro, Phillip Gauger, Yun-Long Tsai, Hwa-Tang Thomas Wang, Pei-Yu Alison Lee

**Affiliations:** 1 Department of Veterinary Diagnostic and Production Animal Medicine, College of Veterinary Medicine, Iowa State University, 1850 Christensen Drive, Ames, IA 50011 USA; 2 National Center for Foreign Animal Diseases, Canadian Food Inspection Agency, Winnipeg, MB Canada; 3 GeneReach USA, Lexington, MA USA

**Keywords:** Seneca Valley virus, SVV, Real-time RT-PCR, Insulated isothermal PCR, RT-iiPCR, POCKIT™

## Abstract

**Background:**

Seneca Valley virus (SVV) has emerged in multiple countries in recent years. SVV infection can cause vesicular lesions clinically indistinguishable from those caused by other vesicular disease viruses, such as foot-and-mouth disease virus (FMDV), swine vesicular disease virus (SVDV), vesicular stomatitis virus (VSV), and vesicular exanthema of swine virus (VESV). Sensitive and specific RT-PCR assays for the SVV detection is necessary for differential diagnosis. Real-time RT-PCR (rRT-PCR) has been used for the detection of many RNA viruses. The insulated isothermal PCR (iiPCR) on a portable POCKIT™ device is user friendly for on-site pathogen detection. In the present study, SVV rRT-PCR and RT-iiPCR were developed and validated.

**Results:**

Neither the SVV rRT-PCR nor the RT-iiPCR cross-reacted with any of the vesicular disease viruses (20 FMDV, two SVDV, six VSV, and two VESV strains), classical swine fever virus (four strains), and 15 other common swine viruses. Analytical sensitivities of the SVV rRT-PCR and RT-iiPCR were determined using serial dilutions of in vitro transcribed RNA as well as viral RNA extracted from a historical SVV isolate and a contemporary SVV isolate. Diagnostic performances were further evaluated using 125 swine samples by two approaches. First, nucleic acids were extracted from the 125 samples using the MagMAX™ kit and then tested by both RT-PCR methods. One sample was negative by the rRT-PCR but positive by the RT-iiPCR, resulting in a 99.20% agreement (124/125; 95% CI: 96.59–100%, κ = 0.98). Second, the 125 samples were tested by the taco™ mini extraction/RT-iiPCR and by the MagMAX™ extraction/rRT-PCR system in parallel. Two samples were positive by the MagMAX™/rRT-PCR system but negative by the taco™ mini/RT-iiPCR system, resulting in a 98.40% agreement (123/125; 95% CI: 95.39–100%, κ = 0.97). The two samples with discrepant results had relatively high C_T_ values.

**Conclusions:**

The SVV rRT-PCR and RT-iiPCR developed in this study are very sensitive and specific and have comparable diagnostic performances for SVV RNA detection. The SVV rRT-PCR can be adopted for SVV detection in laboratories. The SVV RT-iiPCR in a simple field-deployable system could serve as a tool to help diagnose vesicular diseases in swine at points of need.

## Background

Seneca Valley virus (SVV) is a single-stranded, positive-sense RNA virus belonging to the species *Senecavirus A* in the genus *Senecavirus* in the family *Picornaviridae* [1, 2]. Although the species name *Senecavirus A* has been used in some publications as the virus name with an acronym of SVA, in fact the virus name is Seneca Valley virus. The SVV genome (approximately 7.3 kb) contains a single open reading frame (ORF) flanked by a long 5′ untranslated region (UTR; 668 nucleotides) and a short 3′ UTR (68 nucleotides) followed by a poly(A) tail. The polyprotein translated from the single ORF is predicted to be post-translationally processed into four structural proteins (VP4, VP2, VP3, and VP1) and seven nonstructural proteins (2A, 2B, 2C, 3A, 3B, 3C, and 3D) [2].

SVV was initially incidentally identified as a contaminant in PER.C6 cell cultures in 2002 [2]. From 1988 to 2001, a number of virus isolates were sporadically recovered from pigs in various U.S. states but with no detailed description of the clinical symptoms [3]. Sequence analysis of these retrospective virus isolates suggested that these viruses were the same as SVV. Thereafter, SVV was sporadically identified in pigs with swine idiopathic vesicular disease in Canada in 2007 [4] and in the U.S. in 2010 [5], but not much attention was drawn to this virus. At the end of 2014 and the beginning of 2015, multiple outbreaks of vesicular disease in weaned and adult pigs as well as increasing mortality rates of neonatal piglets (1–4 days of age) were reported in Brazil [6–8]. SVV was consistently detected from the pigs with vesicular lesions while other vesicular viral pathogens were not detected [9]. Starting from July 2015, SVV was consistently detected from increasing swine idiopathic vesicular disease cases observed in exhibition, commercial finisher, and breeding swine herds in the U.S. [10]. Foreign animal disease investigations indicated that other vesicular viral pathogens, such as foot-and-mouth disease virus (FMDV), swine vesicular disease virus (SVDV), vesicular stomatitis virus (VSV), and vesicular exanthema of swine virus (VESV), were negative in these cases [10]. Subsequently, SVV detection was reported by other laboratories in the U.S. [11–16], China [17–21], Canada [22], Thailand [23], and Colombia [24]. Vesicular lesions were induced in pigs following experimental inoculation with the contemporary U.S. isolates of SVV [25, 26], confirming that SVV is a vesicular viral pathogen. In one experimental infection study [27], a historical SVV isolate (SVV-001) did not cause overt clinical diseases in the inoculated pigs but it established infection in pigs and induced an immune response. Since the vesicular lesions caused by SVV infection are clinically indistinguishable from those caused by other vesicular disease viruses (e.g., FMDV, SVDV, VSV, and VESV), differential diagnosis is mandatory. RT-PCR is a sensitive and fast method commonly used to differentiate vesicular viral pathogens.

A number of SVV specific gel-based (conventional) RT-PCR, nested RT-PCR, real-time RT-PCR (rRT-PCR), reverse transcription droplet digital PCR, and loop-mediated isothermal amplification assays have been described in the literatures although not all of them have been fully validated [6, 28–35]. Compared to the conventional RT-PCR assays, rRT-PCR is generally more sensitive and suitable for high throughput testing with shorter turnaround time. It is noteworthy that conduction of rRT-PCR assays requires trained technicians and expensive instruments; rRT-PCR assays are mainly performed in the laboratory rather than for on-site applications.

In recent years, a fluorescent hydrolysis probe-based insulated isothermal PCR (iiPCR) technology has been described [36]. The iiPCR and RT-iiPCR can be used for the detection of DNA and RNA molecules. The principle of the iiPCR is to amplify the DNA/RNA by cycling the reaction components through different temperature gradients (denaturation, annealing, and extension) in a capillary tube on a simple Nucleic Acid Analyzer [36, 37]. The iiPCR technology and a commercially available field-deployable device (POCKIT™ combo system), which includes a taco™ mini Automatic Nucleic Acid Extraction System and a POCKIT™ Nucleic Acid Analyzer (GeneReach USA, Lexington, MA, USA), allow automatic detection and interpretation of PCR results within 1–1.5 h [36]. It has been shown that iiPCR or RT-iiPCR assays have excellent sensitivity and specificity for the detection of various targets, including swine pathogens, such as classical swine fever virus (CSFV), FMDV, porcine epidemic diarrhea virus (PEDV), porcine deltacoronavirus (PDCoV), and porcine reproductive and respiratory syndrome virus (PRRSV) [38–41], and various pathogens in shrimp, dogs, cats, poultry, ruminants, and horses [42–52].

In the present study, a SVV rRT-PCR targeting the conserved 5′ UTR and a SVV RT-iiPCR targeting the conserved 3D gene region were developed and validated for the detection of SVV RNA.

## Results

### Genetic diversity of SVV and design of the primers and probes for the SVV real-time RT-PCR and SVV RT-iiPCR

**Fig. 1:**
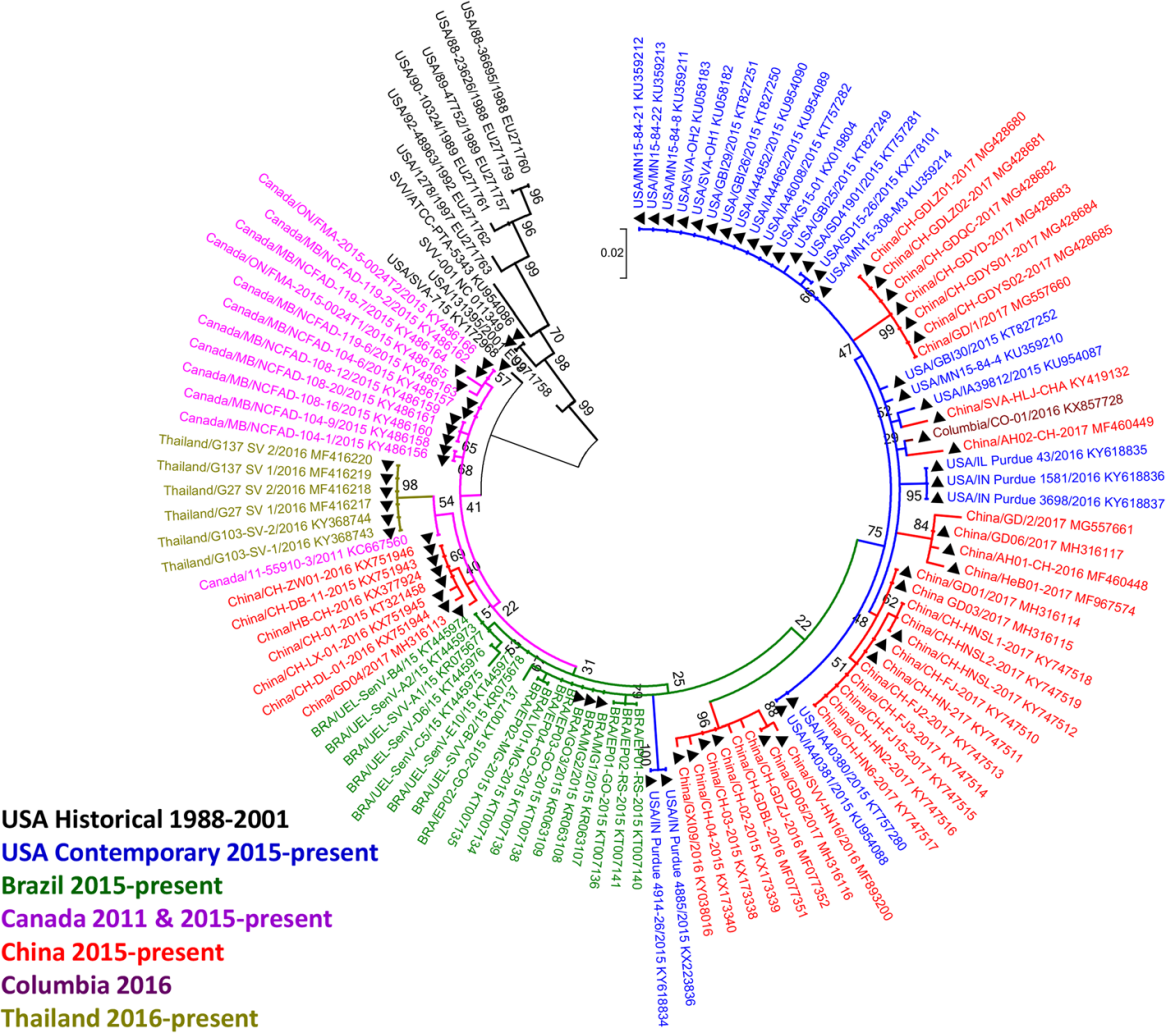
Phylogenetic analysis of global SVV strains. The VP1 sequences of 112 global SVV strains available in GenBank were analyzed to assess the genetic diversity of SVVs. The 79 SVV strains with the whole genome sequences available are denoted with a black triangle. SVV strains from different countries are denoted with different colors. The GenBank accession numbers are included in each strain name

The VP1 sequences are generally used to assess the genetic diversity of SVV. Phylogenetic analyses of the VP1 nucleotide sequences indicated that the global SVV strains formed into the clade of historical SVV (detected in the USA from 1988 to 2001) and the clade of contemporary SVV (detected in the USA, Brazil, Canada, China, Colombia, and Thailand mainly since 2015) (Fig. 1). Based on the VP1 sequences, 112 global SVV strains had 86.2–100% nucleotide identities, with 93.3–99.9% nucleotide identities among the historical clade, 94.2–100% among the contemporary clade, and 86.2–94.8% between the historical and contemporary clades. The 79 SVV strains with whole genome sequences available can well represent the genetic diversity of SVVs (Fig. 1) and these whole genome sequences were used to design the primers and probes targeting the conserved genomic regions. The primers and probe of a SVV rRT-PCR targeting the conserved 5′ UTR and the primers and probe of a SVV RT-iiPCR targeting the 3D gene are shown in Table 1.

**Table 1:** Primers and probes of SVV real-time RT-PCR and SVV RT-insulated isothermal PCR (RT-iiPCR)

Assay name	Primer & Probe	Nucleotide sequence (5′ - 3′)	Nucleotide Position^a^	Target gene	Amplicon
SVV real-time RT-PCR	SVV-F	AACCGGCTGTGTTTGCTAGAG	59–79	5′ UTR	147 bp
	SVV-R	GAACTCGCAGACCACACCAA	205–186		
	SVV-P	6/FAM-CCAAAGGTGTTAGCGCACCCAAACG/IBFQ	143–167		
SVV RT-iiPCR	SVV iiF	GAAGCCATGCTCTCCTACTTCAAA	7033–7056	3D	87 bp
	SVV iiR	TTCTTTTCCAGAATGTTGAGCCA	7119–7097		
	SVV iiP	6/FAM-TCGAGAAGCTGCAATCTG/MGB-NFQ	7070–7086		

^a^Nucleotide positions of SVV primers and probes are based on GenBank accession no. NC_011349

### Analytical specificity of SVV rRT-PCR and RT-iiPCR

As shown in Table 2, the SVV rRT-PCR and RT-iiPCR only specifically reacted with SVV and did not cross-react with any of the vesicular disease viruses that included 20 FMDV strains, two SVDV strains, six VSV strains, and two VESV strains. The SVV rRT-PCR and RT-iiPCR also did not cross-react with the four strains of CSFV and any of 15 other common swine viruses.

**Table 2:** Specificity of SVV real-time RT-PCR (rRT-PCR) and SVV RT-insulated isothermal PCR (RT-iiPCR)

Sample #	Virus	SVV rRT-PCR C_T_	SVV RT-iiPCR Results
1	Seneca Valley virus	16.6486	Pos
2–21	FMDV A24	Neg	Neg
	FMDV A SUD 1	Neg	Neg
	FMDV A22	Neg	Neg
	FMDV SAT2 i	Neg	Neg
	FMDV SAT1 i	Neg	Neg
	FMDV SAT1 ii	Neg	Neg
	FMDV ASIA1 PAK	Neg	Neg
	FMDV C PHI	Neg	Neg
	FMDV SAT3 BEC	Neg	Neg
	FMDV SAT2 SAU	Neg	Neg
	FMDV SAT2 ZIM	Neg	Neg
	FMDV O VIT 7/2006	Neg	Neg
	FMDV ASIA1 PAK 29	Neg	Neg
	FMDV SAT1 BOT 12/2006	Neg	Neg
	FMDV SAT3 SAR 1/2006	Neg	Neg
	FMDV SAT3 UGA 10/97	Neg	Neg
	FMDV C KEN 1/2004	Neg	Neg
	FMDV ASIA1 PAK 20/2003	Neg	Neg
	FMDV O UKG 11/2001	Neg	Neg
	FMDV O MAY 1/2005	Neg	Neg
22–27	VSV NJ UNA 82	Neg	Neg
	VSV IND 85 CLB	Neg	Neg
	VSV IND 94 GUB	Neg	Neg
	VSV NJ 0804 COE3	Neg	Neg
	VSV 0804 COE1	Neg	Neg
	VSV IND	Neg	Neg
28–29	SVDV SWI 1/74	Neg	Neg
	SVDV HKN 1/80	Neg	Neg
30–31	VESV i	Neg	Neg
	VESV C421	Neg	Neg
32–35	CSFV ALFORT 187	Neg	Neg
	CSFV 104	Neg	Neg
	CSFV 906	Neg	Neg
	CSFV 410	Neg	Neg
36	PEDV	Neg	Neg
37	PDCoV	Neg	Neg
38	TGEV	Neg	Neg
39	porcine rotaviruses (A, B, C)	Neg	Neg
40	porcine parvovirus 1	Neg	Neg
41	porcine circovirus 2	Neg	Neg
42	PRRSV-2 (NA type)	Neg	Neg
43	PRRSV-1 (EU type)	Neg	Neg
44	IAV-S H1N1	Neg	Neg
45	IAV-S H3N2	Neg	Neg
46	pHEV	Neg	Neg
47	PRCV	Neg	Neg
48	pseudorabies virus	Neg	Neg
49	porcine teschovirus	Neg	Neg
50	porcine sapelovirus	Neg	Neg
51	PBS (no-template control)	Neg	Neg

*CSFV* Class swine fever virus, *FMDV* Foot-and-mouth disease virus, *IAV-S* Swine influenza A virus, *pHEV* Porcine hemagglutinating encephalomyelitis virus, *PDCoV* Porcine deltacoronavirus, *PEDV* Porcine epidemic diarrhea virus, *PRCV* Porcine respiratory coronavirus, *PRRSV* Porcine reproductive and respiratory syndrome virus, *SVDV* Swine vesicular disease virus, *TGEV* Transmissible gastroenteritis virus, *VESV* Vesicular exanthema of swine virus, *VSV* Vesicular stomatitis virus

### Analytical sensitivity of SVV rRT-PCR and RT-iiPCR

The analytical sensitivities of the SVV rRT-PCR and RT-iiPCR were determined by testing serial dilutions of in vitro transcribed (IVT) RNA. For the SVV rRT-PCR, each dilution of the SVV IVT RNA containing the 5′ UTR (10^8^, 10^7^, 10^6^, 10^5^, 10^4^, 10^3^, 10^2^, 10, 1, 0.1 and 0.01 copies per μL) was run in triplicates. The standard curve had an r^2^ = 0.996 and a slope of − 3.01. The LoD of the rRT-PCR for SVV detection was about 3.5 RNA copies per reaction. For the SVV RT-iiPCR, testing serial dilutions of the IVT RNA containing a fragment of the 3D gene (100, 50, 20, 10, 5, and 0 copies per reaction) revealed that 5/5 (100%), 15/15 (100%), 20/20 (100%), 20/20 (100%), 15/20 (75%), and 0% (0/20) produced positive results on these RNA copies, respectively. The LoD_95%_ of the SVV RT-iiPCR was estimated to be 7 RNA copies per reaction.

The analytical sensitivities of the SVV rRT-PCR and RT-iiPCR were also evaluated by testing RNA extracts from 10-fold serial dilutions (triplicate for each dilution) of SVV cell culture isolates (a US historical isolate SVV-001 and a US contemporary isolate USA/SD41901/2015). The 100% detection endpoints of the SVV rRT-PCR to detect the two SVV isolates were both at 10^− 7^ dilutions (Table 3; 3 TCID_50_ (50% tissue culture infectious dose)/ml). The 100% detection endpoints of the SVV RT-iiPCR was at 10^− 6^ dilutions for the two SVV isolates (Table 3; 30 TCID_50_/ml).

**Table 3:** Analytical sensitivity of SVV real-time RT-PCR (rRT-PCR) and SVV RT-insulated isothermal PCR (RT-iiPCR) using viral RNA from the serially diluted two SVV isolates

Virus strain	Dilution	Theoretical titer (TCID_50_/ml)	SVV RT-iiPCR			SVV rRT-PCR (C_T_)		
			Result 1	Result 2	Result 3	Result 1	Result 2	Result 3
US historical SVV isolate SVV-1 (ATCC-PTA-5342)	10^−4^	3000	+	+	+	28.4	27.8	27.5
	10^−5^	300	+	+	+	31.7	30.8	30.4
	10^−6^	30	+	+	+	34.1	33.8	35.0
	10^−7^	3	?	–	?	36.8	37.3	36.7
	10^−8^	0.3	–	–	–	Neg	Neg	Neg
	10^−9^	0.03	–	–	–	Neg	Neg	Neg
	10^−10^	0.003	–	–	–	Neg	Neg	Neg
US contemporary SVV isolate USA/SD41901/2015	10^− 4^	3000	+	+	+	24.3	25.7	25.6
	10^−5^	300	+	+	+	29.4	29.4	29.3
	10^−6^	30	+	+	+	32.7	32.7	32.7
	10^−7^	3	–	–	–	35.2	36.2	34.3
	10^− 8^	0.3	–	–	–	Neg	Neg	Neg
	10^−9^	0.03	–	–	–	Neg	Neg	Neg
	10^−10^	0.003	–	–	–	Neg	Neg	Neg

TCID_50_, 50% tissue culture infectious dose; C_T_, threshold cycle; Neg, negative

### Performance of SVV rRT-PCR and RT-iiPCR in detecting SVV in clinical samples

The performances of the SVV rRT-PCR and RT-iiPCR for the detection of SVV RNA in swine clinical samples were evaluated by testing 125 clinical samples including 12 vesicular swabs, 30 tonsil swabs, 25 oral fluids, 28 sera and 30 fecal swabs. Distributions of the 125 clinical samples based on specimen types and C_T_ ranges are summarized in Table 4.

**Table 4:** Specimen types of 125 clinical samples and C_T_ ranges of positive samples

Sample type	Number	Positive by the SVV rRT-PCR	
		Number	C_T_ range
Vesicular Swab	12	6	13.2–25.6
Tonsil Swab	30	25	23.6–36.6
Oral fluid	25	6	21.8–35.9
Serum	28	17	15.3–35.7
Fecal swab	30	19	22.5–35.2
Total	125	73	

*rRT-PCR* Real-time RT-PCR

Nucleic acids of the 125 clinical samples were first extracted using a MagMAX™ Pathogen RNA/DNA Kit and then the same nucleic acids were tested by SVV rRT-PCR and RT-iiPCR in parallel with results shown in Table 5. Among the 125 samples, 73 were positive and 52 were negative by SVV rRT-PCR; 74 were positive and 51 were negative by SVV RT-iiPCR. The 73 SVV rRT-PCR-positivie samples were also positive by the RT-iiPCR, while 51 of the 52 rRT-PCR-negative samples were negative by RT-iiPCR. The agreement between the two RT-PCR methods was 99.20% (95% CI: 96.59–101.81%; kappa value = 0.98). Compared to the SVV rRT-PCR, the sensitivity and specificity of the SVV RT-iiPCR were 100 and 98.08%, respectively. The one discrepant sample was retested in triplicate: 1/3 were positive by the rRT-PCR (C_T_ = 33.1) and 2/3 were positive by the RT-iiPCR.

**Table 5:** Performances of SVV real-time RT-PCR (rRT-PCR) and SVV RT-insulated isothermal PCR (RT-iiPCR) on 125 clinical samples extracted by the MagMAX method

		SVV rRT-PCR		Total
		Positive	Negative	
SVV RT-iiPCR	Positive	73	1	74
	Negative	0	51	51
	Total	73	52	125

Sensitivity: 100%; Specificity: 98.08%; Accuracy: 99.20%

Nucleic acid extraction method: MagMAX™ Pathogen RNA/DNA kits on Kingfisher Flex instrument

In the POCKIT™ Combo system, the field-deployable taco™ mini Automatic Nucleic Acid Extraction System (taco™ mini, GeneReach USA, Lexington, MA, USA) allows automatic nucleic acid extraction on site for PCR detection on the POCKIT™ Nucleic Acid Analyzer device. In order to compare the performance of the SVV RT-iiPCR in the POCKIT™ combo system to the SVV rRT-PCR conducted in the laboratory, the following approaches were used. First, nucleic acids were extracted from the 125 clinical samples using the taco™ mini automatic extraction instrument followed by RT-iiPCR testing in the POCKIT™ device (taco™ mini/RT-iiPCR). Meanwhile, nucleic acids were extracted from the same 125 samples using a MagMAX™ Pathogen RNA/DNA Kit followed by the SVV rRT-PCR on the ABI 7500 Fast instrument (MagMAX™/rRT-PCR). As shown in Table 6, among the 125 samples, 73 were positive and 52 were negative by the MagMAX™/rRT-PCR system; 71 were positive and 54 were negative by the taco™ mini/RT-iiPCR system. The 52 MagMAX™/rRT-PCR-negative samples were all negative by the SVV taco™ mini/RT-iiPCR system while two samples positive by the MagMAX™/rRT-PCR system were negative by the taco™ mini/RT-iiPCR system. The agreement between the two systems was 98.4% (95% CI: 95.39–100%; kappa value = 0.97). The two discrepant samples were further tested in triplicate: both samples were positive (3/3) by the MagMAX™/rRT-PCR system (C_T_ = 32.2, 35.7, and 36.0 for one sample; C_T_ = 36.2, 36.6, and 37.9 for another sample), while 0/3 and 1/3 positive by the taco™ mini/RT-iiPCR system, respectively.

**Table 6:** Performances of SVV real-time RT-PCR (rRT-PCR) and SVV RT-insulated isothermal PCR (RT-iiPCR) on 125 clinical samples using different extraction methods and PCR instruments

		MagMAX™/SVV rRT-PCR		Total
		Positive	Negative	
taco™ mini/SVV RT-iiPCR	Positive	71	0	71
	Negative	2	52	54
	Total	73	52	125

Sensitivity: 97.26%; Specificity: 100%; Accuracy: 98.40%

MagMAX™, MagMAX™ Pathogen RNA/DNA kits on Kingfisher Flex instrument; taco™ mini, taco™ Preloaded DNA/RNA Extraction Kit on taco™ mini Automatic Nucleic Acid Extraction System instrument

## Discussion

Recent emergence of SVV infection in multiple countries [6, 10, 11, 17, 23, 24] and the experimental confirmation of SVV as a vesicular viral pathogen [25–27] have raised the concern of differentially diagnosing vesicular diseases in pigs, because the vesicular lesions caused by SVV infection are clinically indistinguishable from those caused by other vesicular disease viruses, such as FMDV, SVDV, VSV, and VESV. The USA is currently free of some foreign animal diseases such as FMDV. Introduction of FMDV would devastate the US pork section. Thus, early detection and recognition of FMDV is critical to minimize the virus spread and economic burden. However, the increased incidence of SVV in the USA may increase the likelihood that veterinarians will assume that the presence of vesicular lesions are due to SVV and not report them to State or Federal animal health officials; this would put the early recognition of FMDV at risk. Therefore, it is critically important to conduct differential diagnosis when vesicular lesions are observed in pigs. RT-PCR is a sensitive and fast method commonly used to differentiate vesicular viral pathogens.

In the current study, we developed and evaluated a SVV rRT-PCR and a field-deployable SVV RT-iiPCR. Both RT-PCR methods are very specific and do not cross-react with other vesicular viral pathogens and other common swine viral pathogens. Both RT-PCR methods can detect the historical and contemporary SVV strains but the SVV rRT-PCR was about 10-fold more sensitive for endpoint detection compared to the SVV RT-iiPCR. The endpoint dilutions negative by the SVV RT-iiPCR but positive by the SVV rRT-PCR had high C_T_ values (34.3–37.3). The SVV rRT-PCR and SVV RT-iiPCR had similar analytical sensitivity in terms of detection in the unit of genomic copies (LoD of 3.5 copies/reaction for SVV rRT-PCR and 7 copies/reaction for SVV RT-iiPCR). Overall, the SVV rRT-PCR and SVV RT-iiPCR had comparable analytical sensitivities.

Subsequently, the diagnostic performances of two SVV RT-PCR assays on clinical samples were evaluated. When the nucleic acids extracted from 125 clinical samples using the MagMAX™ Pathogen RNA/DNA Kit on a Kingfisher Flex instrument were tested, there was only one sample with discrepant results between the SVV rRT-PCR and RT-iiPCR (Table 5). This sample was retested in three replicates, it was found that 1/3 was positive by the rRT-PCR (C_T_ = 33.1) and 2/3 were positive by RT-iiPCR. If the retest results were taken into account, the SVV rRT-PCR and RT-iiPCR would have had zero discrepant results. These data demonstrate excellent agreements between these two SVV RT-PCR methods.

However, for on-site detection, it is impractical to extract nucleic acids from samples using the MagMAX™ Pathogen RNA/DNA Kit on a Kingfisher Flex instrument. Since a portable POCKIT™ Combo package is commercially available that allows on-site nucleic acid extraction using the included taco™ mini instrument and on-site PCR detection using a POCKIT™ Nucleic Acid Analyzer, we further compared the clinical performances of the taco™ mini extraction/RT-iiPCR system to the MagMAX™ extraction/rRT-PCR system based on testing 125 clinical samples. Overall, 98.4% agreement was observed for the two SVV RT-PCR systems (Table 6). When the two discrepant samples were retested in triplicate, three replicates of both samples were positive (3/3) by the MagMAX™/rRT-PCR system (C_T_ = 32.2, 35.7, and 36.0 for one sample; C_T_ = 36.2, 36.6, and 37.9 for another sample), while 0/3 and 1/3 replicates were positive by the taco™ mini/RT-iiPCR system, respectively. Again, these two samples with discrepant results by the two SVV RT-PCR systems had relatively high C_T_ values (low concentrations of virus); sequencing was attempted on these two samples to confirm the RT-PCR results but was unsuccessful. Overall, the data suggest that the SVV MagMAX™/rRT-PCR system and the taco™ mini/RT-iiPCR system have comparable performances on detecting SVV from clinical samples.

In the past a few years, multiple assays have been developed for rapid detection of SVV from clinical samples. These include a conventional two-step RT-PCR assay targeting the VP3/VP1 region [6], a conventional nested-PCR assay targeting the VP1 region [28], a SYBR Green-based real-time RT-PCR assay targeting the VP1 region [29], two TaqMan probe-based real-time RT-PCR assays respectively targeting the 3D region [30] and the VP1 region [31], two reverse transcription droplet digital PCR assays both targeting the 3D region [32, 33], and several reverse transcription loop-mediated isothermal amplification (RT-LAMP) assays targeting the VP1, VP2, 5′ UTR, or VP3/VP1 region [34, 35]. We did not perform a head-to-head comparison of our SVV rRT-PCR and RT-iiPCR to these previously published assays. It is hard to draw a clear conclusion on the analytical sensitivity, diagnostic performance, and cost of all of these described SVV detection assays.

Currently, the samples from pigs with suspect vesicular diseases are submitted to veterinary diagnostic laboratories or the National Veterinary Service Laboratory Foreign Animal Disease Diagnostic Laboratory for differential diagnosis. The sensitive and specific SVV rRT-PCR assay developed in this study can be adopted for SVV detection in laboratories. For the long run, it will be ideal to have some on-site tests capable of quickly and reliably differentiating vesicular viral pathogens so that prompt responses can be taken accordingly without transporting samples, which can be another risk of spreading the virus, to laboratories and waiting for the results. The sensitive and specific SVV RT-iiPCR in a simple field-deployable system described in this study could serve as a tool to help on-site diagnosis of vesicular diseases in swine.

## Conclusions

An rRT-PCR and a field deployable RT-iiPCR were developed and validated for the detection of SVV in this study. Both RT-PCR methods are very specific and do not cross-react with other vesicular viral pathogens and other common swine viral pathogens. Both RT-PCR methods are very sensitive and can detect both the historical and contemporary SVV strains. Furthermore, both RT-PCR systems had comparable diagnostic performances for SVV RNA detection from clinical samples. The SVV rRT-PCR system can be adopted for SVV detection in laboratories. The SVV RT-iiPCR in a simple field-deployable system could serve as a tool to help diagnose vesicular diseases in swine at points of need.

## Methods

Note: Real-time RT-PCR and RT-iiPCR have been previously described for other pathogens. Some procedures for SVV real-time RT-PCR and SVV RT-iiPCR described below are somewhat similar to the methodology previously described for PEDV and PDCoV real-time RT-PCR and RT-iiPCR [40].

### Design of primers and probes for the SVV real-time RT-PCR and SVV RT-iiPCR

The VP1 sequences of 112 global SVV strains available in GenBank were used for phylogenetic analyses to assess the genetic diversity of SVVs. Alignment was conducted using the MUSCLE program and phylogenetic tree was constructed using the maximum-likelihood method of MEGA6 [53] with a bootstrap analysis of 1000 replicates (Fig. 1). The GenBank accession numbers are included in each strain name as shown in Fig. 1. Among the 112 global SVV strains, whole genome sequences were available for 79 SVV strains and they well represented the genetic diversity of SVVs (Fig. 1). Subsequently, the 79 SVV whole genome sequences were aligned and the primers and probes were designed to target the conserved genomic regions using the Primer Express software 3.0.1 (Thermo Fisher Scientific). Specifically, a SVV rRT-PCR targeting the conserved 5′ UTR and a SVV RT-iiPCR targeting the 3D gene were developed in this study (Table 1).

### Viruses

A U.S. historical SVV isolate (SVV 001, ATCC-PTA-5342) obtained from the American Type Culture Collection (Manassas, VA, USA) and a U.S. contemporary SVV isolate USA/SD41901/2015 obtained at the Iowa State University Veterinary Diagnostic Laboratory (ISU VDL) [10] were used for evaluating the analytical sensitivities of the SVV RT-PCRs. Both SVV isolates were propagated and titrated in H1299 cell line obtained from the American Type Culture Collection (ATCC CRL-5803). For analytical sensitivity analysis, the two SVV cell culture isolates with infectious titers of 10^7^ TCID_50_/ml were 10-fold serially diluted in minimum essential medium and subjected to RNA extraction and RT-PCR testing in triplicate for each dilution.

Twenty FMDV strains, two SVDV strains, six VSV strains, two VESV strains, and four CSFV strains (Table 2) were used for evaluating the analytical specificities of the SVV RT-PCRs in the National Centre for Foreign Animal Disease (NCFAD) located at Winnipeg, Canada. The FMDV and SVDV isolates were obtained by NCFAD from the World Reference Laboratory for FMD, Pirbright Institute, UK which usually receives FMDV submissions from affected countries. At the NCFAD, FMDV and SVDV isolates were propagated in baby hamster kidney (BHK-21, ATCC CCL-10) and porcine kidney (IBRS2, ATCC CRL-1835) cell lines, respectively. The VSV isolates were obtained from Plum Island Animal Disease Center, Greenport, NY, USA and amplified in African green monkey (Vero, ATCC CCL-81) cells at the NCFAD. The CSFV and VESV isolates were obtained by the NCFAD respectively from the World Reference Laboratory for CSF in Hannover, Germany and Animal Disease Research Institute, Nepean, Canada and each was amplified in porcine kidney (PK-15, CCL-33) cells. Nucleic acid was extracted from the cell culture supernatants for specificity testing.

Additional swine viral pathogens used for evaluating analytical specificities of the SVV RT-PCRs included PEDV, PDCoV, transmissible gastroenteritis virus, porcine rotaviruses (A, B, C), porcine parvovirus 1, porcine circovirus 2, PRRSV-1, PRRSV-2, swine influenza A virus (H1N1 and H3N2), porcine hemagglutinating encephalomyelitis virus, porcine respiratory coronavirus, pseudorabies virus, porcine teschovirus, and porcine sapelovirus (Table 1). All of these viruses were available at the ISU VDL and were grown in the appropriate cell lines obtained from ATCC. Phosphate buffered saline (PBS) was included as a negative control. Nucleic acid was extracted from these viruses and PBS for specificity testing.

### Clinical samples

A total of 125 swine clinical samples (12 vesicular swabs, 30 tonsil swabs, 25 oral fluids, 28 sera, and 30 fecal swabs) collected from various states within the U.S. since 2015 were used to evaluate the diagnostic performances of SVV RT-PCRs. All of these samples were submitted by veterinarians to the Iowa State University Veterinary Diagnostic Laboratory for routine testing.

### Nucleic acid extraction

Nucleic acids were extracted from various virus isolates, vesicular swabs and sera using a volume of 50 μL of samples as well as from tonsil swabs, oral fluids and fecal swabs using a volume of 100 μL of samples. Nucleic acid extraction was conducted using a MagMAX™ Pathogen RNA/DNA Kit (Thermo Fisher Scientific, Waltham, Massachusetts, USA) on a Kingfisher-Flex instrument (Thermo Fisher Scientific) following the instructions of the manufacturer. Nucleic acids were eluted into 90 μL of Elution buffer.

Nucleic acids were also extracted from clinical samples (vesicular swabs, tonsil swabs, oral fluids, sera and fecal swabs) using taco™ Preloaded DNA/RNA Extraction Kit on a taco™ mini. Briefly, 200 μl of the samples were added into the first wells of an extraction plate which was subsequently placed into the device following the manufacturer’s user manual. Nucleic acids were eluted into 200 μl of Elution buffer.

### In vitro transcribed (IVT) RNA

To prepare the SVV RNA standards, plasmids containing a fragment of 5′ UTR or 3D region of SVV USA/SD41901/2015 (GenBank accession no. KT757281) were synthesized (IDT, Coralville, Iowa, USA). The plasmids were linearized, purified and subjected to run-off in vitro transcription into RNA using a MEGAscript T7 Transcription Kit (Thermo Fisher Scientific). RNA transcripts were produced, treated with Turbo DNase, and purified using the MEGAclear™ Transcription Clean-Up Kit (Thermo Fisher Scientific) following the manufacturer’s instructions. Copy numbers of RNA transcripts were calculated based on concentrations determined by a NanoDrop 2000 spectrophotometer (*Thermo* Fisher Scientific). Serial dilutions of RNA were prepared in nucleic acid dilution solution. Aliquots were frozen at − 80 °C for single use of each aliquot.

### SVV real-time RT-PCR and SVV RT-iiPCR

SVV rRT-PCR was set up in a 20 μL total reaction using TaqMan® Fast 1-Step Master Mix (Thermo Fisher Scientific): 5 μL of 4× Master Mix, 0.4 μL of forward primer at 20 μM, 0.4 μL of reverse primer at 20 μM, 0.24 μL of probe at 10 μM, 1 μL XENO Internal Positive Control Reagent (Thermo Fisher Scientific), 7.96 μL nuclease-free water, and 5 μL nucleic acid extract. Amplification reactions were performed on an ABI 7500 Fast instrument (Thermo Fisher Scientific) with the following conditions: 1 cycle of 50 **°**C for 5 min, 1 cycle of 95 **°**C for 20 s, and 40 cycles of 95 **°**C for 3 s and 60 **°**C for 30 s. Any cycle threshold (C_T_) value < 40 was reported as positive.

The POCKIT™ SVV Reagent Set (GeneReach USA) was a lyophilized TaqMan probe-based RT-PCR reaction. The Premix was reconstituted with 50 μL Premix Buffer B before the addition of 5 μL nucleic acid, and 50 μL of the mixture was transferred to an R-tube™ (GeneReach USA). The tube was spun briefly in a cubee™ microcentrifuge and placed into a POCKIT™ Nucleic Acid Analyzer for RT-PCR reaction. The default program in the POCKIT™ device converted automatically the signal-to-noise (S/N) ratio to positive “+” or negative “-” [36] and display them on the screen after the reaction is completed. Based on the default thresholds, S/N ratios of < 1.2 and > 1.3 were assigned as “+” and “−”, respectively. A “?” result was assigned to those with an S/N ratio between 1.2 and 1.3, indicating that the signals were ambiguous and the sample should be retested again.

### Statistical analyses

Limit of detection 95% (LoD_95%_) was determined by probit analysis with a 95% confidence interval (95% CI) by using the SPSS v14 (SPSS, Chicago, IL, USA). Kappa analyses was used to assess interrater agreement.

## Data Availability

The data set(s) supporting the results of this article are included within the article.

## Abbreviations

CSFV: Classical swine fever virus
C_T_: Cycle threshold
FMDV: Foot-and-mouth disease virus
IVT: In vitro transcribed
LoD: Limit of detection
PDCoV: Porcine deltacoronavirus
PEDV: Porcine epidemic diarrhea virus
PRRSV: Porcine reproductive and respiratory syndrome virus
rRT-PCR: Real-time RT-PCR
RT-iiPCR: Reverse transcription insulated isothermal PCR
RT-PCR: Reverse transcription polymerase chain reaction
SVDV: Swine vesicular disease virus
SVV: Seneca Valley virus
TGEV: Transmissible gastroenteritis virus
UTR: Untranslated region
VESV: Vesicular exanthema of swine virus
VSV: Vesicular stomatitis virus
